# DETECT-DVT: Detroit Evaluation of Thrombectomy and Evaluation of Intravascular Ultrasound in Deep Vein Thrombosis

**DOI:** 10.1016/j.jscai.2024.102153

**Published:** 2024-09-20

**Authors:** Sabina Kumar, Brian Ballard, Umeh Chukwuemeka, Anthony Teta, Mustafa Turkmani, Anuraag Khandavalli, Samuel Reenders, Arjun Chadha, Marian Canon, Saman Barznji, Jason Kaplan, Varun Yelamanchilli, Brandon Ballard, Mark Zainea, Jay Mohan

**Affiliations:** a Division of Cardiology, Michigan State University, East Lansing, Michigan; b McLaren Cardiovascular Institute, Macomb, Michigan; c Department of Cardiology, Hemet Global Medical Center, Hemet, California; d Department of Surgery, Mayo Clinic, Rochester, Minnesota

**Keywords:** deep vein thrombosis, intravascular ultrasound, mechanical thrombectomy, venous disease

## Abstract

**Background:**

We sought to evaluate the use of intravascular ultrasound (IVUS) and mechanical thrombectomy (MT) for the treatment of deep vein thrombosis (DVT) in a community hospital setting.

**Methods:**

Data were analyzed among patients with lower extremity DVT who underwent MT from December 1, 2021 to December 1, 2022.

**Results:**

A total of 1263 patients were evaluated and only 8.8% of patients with DVT received intervention. Of them, 42% were women. The mean age and length of stay were 61.3 years and 3.5 days, respectively. For cases that proceeded to intervention, IVUS was used in 89% of cases, 80% received venoplasty, and 30% received stents. The mean number of MT passes was 4 and the mean contrast volume used was 71 mL. Flow was restored in 96.7% of cases. The procedure was unable to be completed in 1.8% of the cases, and 1.8% had a reported complication after the procedure. Vascular surgery was consulted in 64.4% of the cases, cardiology in 33%, interventional radiology in 12.5%, and 10.9% of the patients had multiple consults. MT was associated with postprocedure reduction of hemoglobin levels (13.4 vs 12.1; *P* < .001) and no change in postprocedure creatinine levels (1.08 vs 1.04; *P* = .28). IVUS was associated with fewer passes, although this was not statistically significant (*P* = .09). Additionally, IVUS was associated with increased stenting (*P* = .03) and venoplasty (*P* < .001).

**Conclusions:**

MT is shown to be successful in restoring venous flow and is utilized by multiple specialties in the treatment of DVT. Additionally, IVUS was widely used in conjunction with MT, and it was associated with increased advanced interventions, such as venoplasty and stent placement.

## Introduction

Deep vein thrombosis (DVT) affects 1 in 1000 adults annually and continues to pose a significant public health challenge worldwide. While DVT itself can cause regional complications, its greatest threat lies in its potential to embolize, leading to potentially life-threatening pulmonary embolism (PE) and chronic thromboembolic pulmonary disease. One-third of all DVT, primarily in the proximal location, will progress to PE, carrying substantial clinical consequences. Hence, venothromboembolic disease (DVT and PE) is the third leading cause of cardiovascular death behind myocardial infarction and stroke.^1^

The estimated health care costs for venous thromboembolism (VTE) in the United States are as high as 10 billion dollars annually.^2^ About 33% of patients with VTE will have a recurrence in 10 years, 33% to 50% will develop postthrombotic syndrome (PTS), and >50% of working-age individuals will have some disability.^3^^,^^4^ The standard therapy for VTE is 3 to 6 months of oral anticoagulation (OAC). However, OAC does not successfully treat all forms of clots (ie, chronic thrombus) and, therefore does not prevent the sequelae of PTS and recurrence of DVT. PTS has been reported to occur in as high as 40% to 60% of patients with DVT and^3^^,^^4^ is primarily diagnosed 6 months after the initial event. Due to our current deficiencies in the prevention of PTS, there has been a paradigm shift from OAC to catheter-based interventional therapies. Modern operators are now electing to escalate care to invasive catheter-based approaches to help alleviate patient’s symptoms.

Recent advances in mechanical thrombectomy (MT) have become an option for the treatment of DVT and PE. Research has progressed starting with CaVent and ATTRACT looking at outcomes with catheter-directed thrombolysis (CDT) vs OAC to treat DVT. Following this CAVA, Bernitiful, and ACCESS looked at outcomes with ultrasound-assisted thrombolysis vs OAC. Then more recently, thrombectomy registry data, CLOUT, has reviewed the potential benefits of MT.

Additionally, with the progression to catheter-based therapies for the treatment of DVT, the utilization of intravascular imaging in the venous space continues to improve. In this context, imaging continues to play an important role in the intraoperative assessment of venous disease. Intravascular ultrasound (IVUS) has allowed us to better understand anatomical variants leading to complex venous disease as well as an objective way to assess outcomes of catheter-based interventions.

However, there are no randomized controlled trials for MT, and thus, a lack of clear-cut widely accepted guidelines for the use of MT in the treatment of DVT. Therefore, it is imperative to develop research regarding interventional treatment modalities for DVT, which may address the high occurrence of postthrombotic syndrome, life-threatening PE, and long-term chronic thromboembolic pulmonary disease in these patients.

## Materials and Methods

### Study design and population

This is a retrospective chart review of patients who presented to the emergency department (ED) with newly diagnosed DVT in 4 community hospitals in Michigan between December 2021 and December 2022. A total of 1557 patients were initially identified, and upon further review, 294 patients met the exclusion criteria and 1263 patients met the inclusion criteria (Central Illustration). The inclusion criteria were all patients admitted to the ED with a proximal lower extremity DVT (thrombus involving the inferior vena cava [IVC], iliac, common femoral, femoral, and popliteal veins) or a distal lower extremity DVT (thrombus involving the calf veins, which include peroneal, posterior, anterior tibial, and gastrocnemius veins). The exclusion criteria were patients who were aged <18 years, left against medical advice, were diagnosed with a DVT on a prior admission, were not diagnosed in the ED, or had a DVT in vessels other than the lower extremities.

**Central Illustration:**
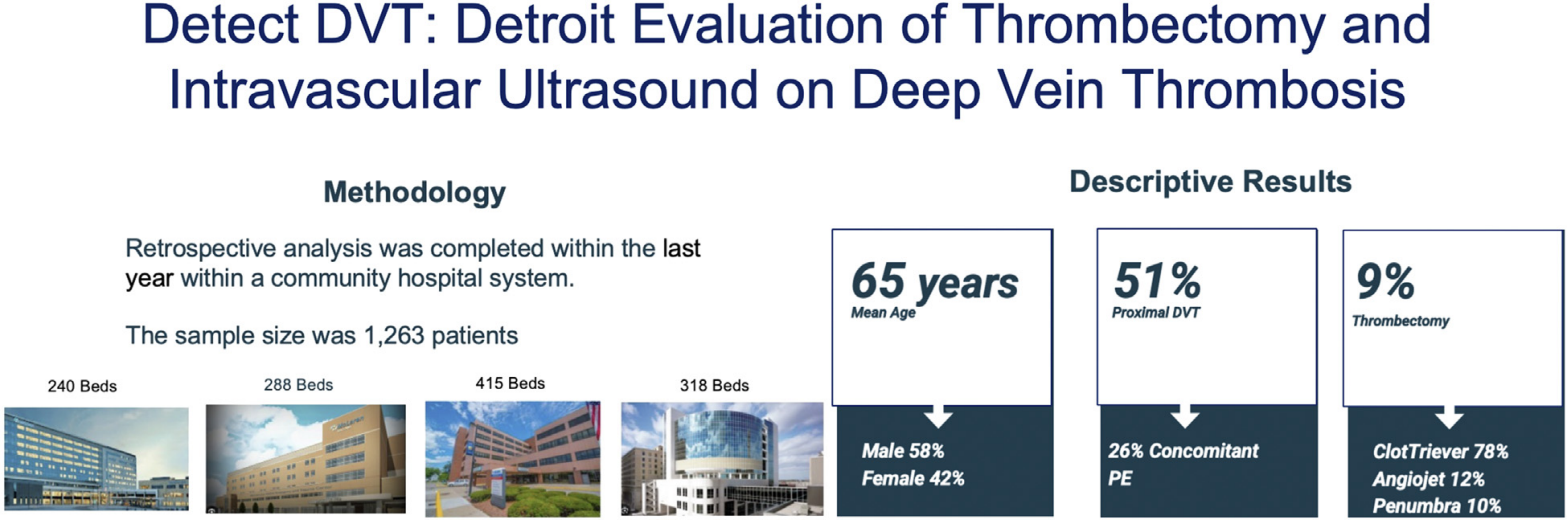
The methodology and the descriptive statistics for our project DETECT-DVT: Detroit Evaluation of Thrombectomy and Intravascular Ultrasound on Deep Vein Thrombosis. DVT, deep vein thrombosis; PE, pulmonary embolism.

### Data source and extraction

Relevant data were anonymized and extracted from eligible patients’ electronic medical records (EMR) using International Classification of Disease (ICD) codes for DVT. This data included patients’ demographic characteristics, comorbidities, location, acuity of DVT, laboratory test results on admission, presence of concomitant PE, and treatment received for DVT. Data extraction adhered to institutional anonymization protocols and was conducted by physicians. The definition of an acute DVT was symptom onset of 2 weeks, subacute DVT 2 to 4 weeks, and chronic DVT to >4 weeks. Ultrasound imaging was used to determine chronicity of the DVT and the history and physical were used to augment the above definition.

### Variables and measurements

The primary outcome variables were factors associated with both MT and IVUS utilization in patients with DVT.

### Statistical analysis

Univariate analysis of DVT patient characteristics and the subgroup receiving MT employed descriptive statistics (means and percentages). Bivariate analysis compared groups using χ^2^ tests for categorical variables and *t* tests for continuous variables. A backward selection logistic regression model identified independent predictors for both MT and IVUS use. Biologically plausible and statistically significant variables from the bivariate analysis, such as age, gender, PE presence, and DVT laterality and acuity, were included as independent variables in the final model. Statistical analyses were performed using SPSS version 27.0 (IBM Corp).

## Results

There were 1263 patients, of which 45.3% were women and 87.3% were White. The mean age was 65 years (SD 14.6), and the mean length of hospital stay was 3.4 days. Of the patients with DVT, 51.9% had proximal DVT, 83.8% had unilateral DVT; 26.3% had concomitant PE, 61.5% had acute DVT, and 38.5% had a subacute or chronic DVT. In all the patients with DVT, left-sided, right-sided, and bilateral involvement were 44.8%, 39.5%, and 15.7%, respectively. Similarly, in patients with proximal DVT, left-sided, right-sided, and bilateral involvement were 47.1%, 33.7%, and 19.3%, respectively. Furthermore, in the patients with distal DVT left-sided, right-sided, and bilateral involvement were 67.8%, 26.7%, and 5.6%, respectively. Further, 39.6% had a prior history of DVT, 48% had a provoked DVT, and 8.8% had an MT for DVT (Table 1).

**Table 1:** Characteristics of patients with DVT in the study population.

Characteristic	N = 1263
Mean age, y	65 ± 14.6
Mean length of hospital stay, d	3.4 ± 5.5
Female sex	45.3%
Race	
White	87.3%
African American	12.3%
Other	0.4%
Patients with concomitant PE	26.3%
DVT location	
Proximal	51.9%
Distal	48.1%
DVT laterality	
Unilateral	83.8%
Bilateral	16.2%
DVT chronicity	
Acute	61.5%
Subacute and chronic	38.5%
Proportion that received mechanical thrombectomy for DVT	8.8%
Proportion that received mechanical thrombectomy for PE	4.3%

Values are mean ± SD or %.

DVT, deep vein thrombosis; PE, pulmonary embolism.

MT for DVT was more likely to be performed in younger patients, those with unilateral DVT, and those with acute DVT. Our analysis showed decreased odds of MT with increasing age (odds ratio [OR], 0.98; 95% CI, 0.971-0.997; *P* = .016), bilateral DVT (OR, 0.45; 95% CI, 0.211-0.936; *P* = .033), and subacute and chronic DVT (OR, 0.57; 95% CI, 0.357-0.909; *P* =.018) (Table 2).

**Table 2:** Factors associated with mechanical thrombectomy in patients with DVT.

Factors	*P* value	Odds ratio	95% CI
Age	.016	0.984	0.971-0.997
Unilateral vs bilateral DVT	.033	0.445	0.211-0.936
Acute vs subacute and chronic DVT	.018	0.57	0.357-0.909

Values are mean ± SD or %.

DVT, deep vein thrombosis.

In the subanalysis of patients who received MT, 42% were women. The mean age and length of stay were 61.3 years and 3.5 days, respectively. IVUS was used in 89% of cases, 80% received venoplasty, and 30% received stents; 28.9% returned for further intervention after the initial procedure. The mean number of MT passes was 4, and the mean contrast volume used was 71 mL. Flow was restored in 96.7% of cases. Of the patients, 1.6 % had a complication. Vascular surgery was consulted in 64.4% of the cases, cardiology in 33%, and interventional radiology in 12.5%. Of the patients, 10.9% had multiple consults (Table 3).

**Table 3:** Characteristics of patients who received thrombectomy.

Characteristic	N = 111
Mean age, y	61.3 ± 14.3
Female sex	42%
Proximal DVT	100%
Unilateral DVT	94.0%
Acute DVT	75.3%
Subacute and chronic DVT	24.7%
Prior history of DVT	35.9%
Provoked DVT	51.0%
History of IVC filter	8.2%
Consults for DVT treatment	
Multiple consults	10.9%
Cardiology	33.0%
Vascular surgery	64.4%
Interventional radiology	12.5%
Mechanical thrombectomy	
IVUS used	89%
Complications	1.8%
Flow restored	96.7%

Values are mean ± SD or %.

DVT, deep vein thrombosis; IVUS, intravascular ultrasound.

In the bivariate analysis, IVUS use was associated with increased stenting (*P* = .03), increased venoplasty (*P* < .001), and decreased complications (*P* < .001). In the multivariate analysis, IVUS use was associated with increased odds of venoplasty (OR, 8.25; 95% CI, 2.02-33.68; *P* = .003).

Additionally, the use of IVUS was associated with fewer passes, although this was not statistically significant (*P* = .09) (Table 4). IVUS was used in all the patients with ≤4 passes.

**Table 4:** Bivariate analysis of the relationship between categorical variables and IVUS use.

	Intravascular ultrasound		
Variable	No	Yes	*P* value
Sex			.652
Male	9.8%	90.2%	
Female	12.8%	87.2%	
Stenting			.027
No	16.1%	83.9%	
Yes	0.0%	100.0%	
Venoplasty			<.001
No	33.3%	66.7%	
Yes	5.7%	94.3%	
Flow restored			.218
No	33.3%	66.7%	
Yes	10.5%	89.5%	
Complications			<.001
No	5.3%	94.7%	
Yes	100.0%	0.0%	
No. of passes			.091
≤4 passes	0.0%	100.0%	
>4 passes	11.1%	88.9%	

Safety outcomes following MT were generally favorable with 2 patients with all-cause mortality, 0 device-related adverse events, 1 access site event, and 1 cerebrovascular accident (CVA). In 2 patients, the procedure was aborted. MT was associated with postprocedure reduction of hemoglobin levels (13.4 vs 12.1; *P* < .001). There was no change in postprocedure creatinine levels (1.08 vs 1.04; *P* = .28) (Table 5).

**Table 5:** Safety outcomes for mechanical thrombectomy.

Safety outcomes	Number of patients
All-cause mortality	2
Serious adverse events	0
Pulmonary embolism	0
Device-related	0
Cardiac arrest	0
Epistaxis	0
Pulseless electrical activity	0
Respiratory failure	0
Cerebrovascular accident	1
Access site	1
Postoperative bleeding event	0
Procedure aborted	2

## Discussion

Several trials have shaped the current landscape in DVT treatment. The CaVenT study,^5^ a landmark trial comparing CDT to OAC, demonstrated a 14.4% absolute risk reduction in PTS at 24 months but with an increased risk of bleeding. The ATTRACT trial^5^ evaluated CDT, finding no significant difference in PTS outcomes at 24 months, alongside a rise in major bleeding events.

For ultrasound-assisted thrombolysis, both the CAVA study^6^ and the Bernutiful study^7^ failed to show significant outcome differences in PTS compared with standard CDT. However, ACCESS PTS^8^ reported a substantial reduction in PTS severity in patients with chronic DVT undergoing this treatment, suggesting its potential in managing chronic-phase patients.

Recent developments in MT are highlighted by the CLOUT^9^ and BOLT registries. The CLOUT registry, examining the efficacy of the Inari Clotriever, has shown promising results with a significant reduction in clot burden.^9^ The BOLT registry is currently investigating the Penumbra Indigo aspiration system, with enrollment still underway.

DVT management primarily adheres to societal guidelines that serve as frameworks for practitioners globally. However, these guidelines have yet to fully integrate newer interventional methods, particularly MT, leaving a void in comprehensive treatment strategies.

The National Institute of Health and Care Excellence guidelines from the United Kingdom, updated in June 2019, recommend anticoagulation as the primary treatment for DVT, with MT reserved for specific scenarios involving proximal DVT or for research purposes in distal DVT cases not extending into the common femoral vein.^10^

The American College of Chest Physicians, in their 2021 guidelines, advocates for anticoagulation over interventional therapy in acute DVT cases. They cite a lower incidence of PTS and increased bleeding risks associated with interventional treatments, although they note the potential benefits of rapid thrombus resolution in severe DVT cases.^11^ The American Society of Hematology also supports OAC as the primary DVT treatment, suggesting CDT in specific scenarios.^12^

The European Society of Vascular Surgery provides insights into interventional methods, indicating that anticoagulation alone may not suffice for PTS prevention. They report noninferiority of surgical thrombectomy to thrombolysis for PTS management at the 2-year mark and express a preference for CDT performed in a pulsatile fashion to avoid complications, such as valvular dysfunction.^13^

In recent years, there has been a notable increase in the adoption of interventional catheter-based therapies to immediately manage acute DVT to improve long-term outcomes. These therapies, encompassing CDT, MT, venoplasty, and venous stenting, offer the potential for improved patient outcomes.^14^ Our study had 1263 patients with DVT; however, only 8.8% of patients received an MT. This could be due to physician bias based on the fact there are no clear-cut universally accepted guidelines.

There have been recent advancements in MT, and among the notable advancements are the AngioJet (Boston Scientific), ClotTriever System (Inari Medical), and Lightning Indigo (Penumbra). The AngioJet system, initially designed for thrombectomy in coronary and peripheral vessels, combines mechanical fragmentation, pharmacologic lysis, and rheolytic aspiration. The ClotTriever System, an MT device, effectively removes thrombus from veins in both acute and chronic DVT cases.^9^^,^^15^ The Lightning Indigo system is a vacuum-assisted MT that removes thrombus from both veins and arteries of various sizes. Within the patients in our study population that received an MT, 78.2% were with the ClotTriever, 12% with the AngioJet, and 9.8% with the Lightning Indigo. The mean number of passes in patients with ClotTriever and Penumbra were 3.81 vs 6.0 respectively. The small sample size within the Lightning Indigo group may limit the above subgroup analysis; therefore, we recommend a larger sample size to adequately distinguish the different treatment modalities and the number of passes.

In addition to MT, CDT is a viable option for managing complex venous obstruction. CDT allows for direct delivery of thrombolytic agents to the target lesion resulting in substantially lower doses of the thrombolytic agent used and decreased rates of intracranial bleeding complications, in contrast to the higher rates associated with systemic administration.^9^ In our study, 10% of patients received CDT and 1 patient had both CDT and MT. IVUS was not used in any of the patients who had CDT.

In the past, CDT represented the most common interventional treatment option when intervening on DVT; however over the past decade, MT has emerged as a new therapeutic option for DVT. The thrombectomy device market is rapidly expanding and is projected to grow from $1.34 billion in 2022 to $1.95 billion by 2027.^16^^,^^17^ North America is the largest consumer, with the Asia Pacific region poised as the fastest-growing market. In the first quarter of 2023, Inari Medical led with a 51.5% market share, followed by Penumbra, Boston Scientific, and AngioDynamics.^18^

Furthermore, our study found MT was more likely to be performed in younger patients and those with unilateral DVT. This could be a possible limitation of the data as physician bias may have contributed to which patients received an MT. This finding was similar in other studies, such as CLOUT, in which 95.6% of patients had unilateral DVT and 4.4% had bilateral DVT.^9^ Similarly, our study showed that having an acute DVT was a predictor of MT utilization. This is similar to the CLOUT registry, whose population had 63% subacute or acute DVT and 35.8% chronic DVT. The ATTRACT trial, a study that focused on the impact of additional pharmacomechanical CDT in minimizing PTS, was only limited to patients with acute DVT, excluding those with subacute or chronic DVT.^19^

Additionally, our data revealed that most of the consultations for DVT patients were for vascular surgery, followed by cardiology, and interventional radiology. Decisions regarding DVT management, including consultations, are often individualized and institution-dependent. As endovascular procedures have led to improved clinical outcomes (including risks of infection, duration of hospital stay, and quicker recovery times) than open surgery, concerns have been cited regarding the territorial conflict in managing vascular pathologies.^20^ Notably, internists are more likely than surgeons to refer endovascular procedures to interventional cardiology or interventional radiology over vascular surgery.^21^ IVUS was used in 89% of patients with a cardiology consult, 91% in those with an interventional radiology consult, and 84% in those with a vascular surgery consult. Data regarding consultation patterns with DVT management are limited. We suspect there is high variability in the consultation patterns of DVT management across different institutions, but further research is required.

Safety outcomes were generally favorable (Table 5). The 2 patients in the all-cause mortality group both elected for hospice care due to malignancy and after severe disability due to an intraparenchymal hemorrhage after a fall. The patient diagnosed with the CVA underwent an IVC thrombectomy and postoperatively, was found to have a right atrium thrombus. It is unclear if the CVA was related to the patient’s venous thrombus; however, it is plausible that the patient had an undiagnosed right to left intracardiac shunt such as a patent foramen ovale or atrial septal defect. The patient with the access site complication did require vascular consultation; however, no further intervention was needed. No bleeding events were associated with the need for packed red blood cell transfusion. In the 2 patients where the procedure was aborted, anatomical technical challenges precluded the ability to intervene specifically due to the inability to cross a complete left common iliac occlusion and the other due to the inability to cross a previous ligation of the iliac vein from a motor vehicle accident that was discovered postoperatively.

Further, 28.9% of patients were brought back for repeat procedures, most of which involved treatment of proximal vein stenosis with venoplasty and stenting. There are no current guidelines suggesting an optimal time frame for staged procedures in venous interventions. Additionally, it is unclear if certain venous lesions (ie, venous compression) should be treated at the time of the index procedure or in a staged fashion. This likely is multifactorial depending on the interventionist, the compliance of the patient taking OAC, the patient's anatomy on IVUS, and the amount of contrast used for the thrombectomy portion. Additionally, there is some concern that venous lesions may appear more severe based on patient positioning (ie, supine vs prone) suggesting that patients may benefit from staged procedures to optimize positioning during each intervention. Due to these concerns and the lack of consensus, it is imperative to further research in this area.

Our study showed a high use of IVUS in DVT MT cases, with IVUS being used in 89% of cases. The high usage of IVUS in our MT patients likely reflects the practice patterns of the operators who were more likely to use MT as an early treatment approach. This seems to reflect current trends nationally with the use of IVUS in MT, specifically in interventional cardiology operators.

IVUS has emerged as a pivotal imaging modality in managing venous disease, especially in patients with DVT. IVUS provides real-time circumferential images, enabling precise thrombus extent and characterization, limits contrast and radiation usage, and gives accurate details regarding venous anatomy assessment and reasons for venous stenosis compared to venography alone (Table 6).^22^

**Table 6:** Characteristics of IVUS vs venography.

Characteristics	IVUS	Venography
Stenosis	+++	++
Plaque morphology	+++	+
Acute thrombus	++	+
Chronic thrombus	+++	+
Residual thrombus	+++	+
Flow	–	+++
Extrinsic and dynamic compression	+++	+
Identification of landing zones of stent	+++	++
Stent expansion	+++	++
Stent apposition	+++	+
Dissection	+++	++
Contrast needed	–	Yes
Radiation needed	–	Yes

Adapted, updated, and reprinted with permission from Secemsky et al,^22^ 2022.

+ Fair; ++ Good; +++ Excellent; − Not applicable; IVUS, intravascular ultrasound.

Murphy et al^23^ performed a prospective single-arm study of 33 patients undergoing CDT with IVUS preintervention and postintervention. IVUS compared to venography alone was able to identify stenosis, residual thrombus, and May-Thurner anatomy requiring further intervention. Raju et al^24^ presented a cohort of 65 patients undergoing CDT for DVT and found that IVUS was more sensitive than venography in detecting residual thrombus. Residual thrombus can lead to PTS, PE, and recurrent DVT leading to the importance of IVUS utilization in venous interventions. This is reflected in our study where IVUS usage was associated with decreased passes during MT. IVUS was used in all the MT patients with ≤4 passes.

Intravascular ultrasound does not require contrast or radiation as compared to venography. Alhalboun et al^25^ have progressed to utilizing IVUS alone in patients with renal insufficiency for the treatment of venous obstruction. In our study, the low volume of contrast usage (71 mL) was possibly attributed to 89% of IVUS use for MT cases. There was also no change in postprocedure creatinine levels in our study population (1.08 vs 1.04; *P* = .28).

Intravascular ultrasound use in our study was associated with increased stenting, increased venoplasty, and decreased complications. IVUS-guided venous stenting has demonstrated improved patency rates and clinical outcomes. Tran et al^26^ found that IVUS examination before stent deployment was associated with larger stent implants and lower repeat revascularization at 30 days and 2 years when compared with venography.

Intravascular ultrasound enhances the accuracy in assessing preintervention and postintervention venous lesion characteristics and facilitating precise stent sizing and placement. This accuracy is crucial for reducing symptom recurrence and repeat interventions. The VIDIO (Venogram vs Intravascular Ultrasound for Diagnosing and treating Iliofemoral Vein Obstruction) study, which was a multicenter prospective single-arm study (n = 100), demonstrated that IVUS was more sensitive than venography in identifying venous stenosis; it identified 26.3% significant lesions that were missed by venography and changed the treatment plan in 57% of patients.^27^ Neglén et al^28^ compared IVUS with venography in 304 venous interventions and found that on average, the transfemoral venography significantly underestimated the degree of stenosis by 30%. Venography was inaccurate in the detection of obstruction of >70% as compared with IVUS.

Intravascular ultrasound guides the procedure in venous stenting and plays a crucial role in postprocedural assessments. The enhanced visualization of the stent apposition and expansion provided by IVUS correlates with lower rates of stent migration and thrombosis. Montminy et al^29^ presented a retrospective study of 155 limbs with chronic vein occlusions treated with IVUS-associated stenting between 2013 and 2015. The study found that venography was inferior to IVUS in determining optimal proximal and distal landing zones for venous stenting, which could lead to stent malapposition and need for further reintervention. In addition, an analysis of retrospective data on Medicare beneficiaries undergoing DVT intervention between 2017-2019 indicated that IVUS-guided stenting was associated with a reduced rate of stent migration, reintervention, or rehospitalization over a 12-month follow-up.^30^

Despite the benefits of IVUS as an adjunct to venous interventions, the lack of large randomized control trials in the venous space comparing IVUS with venography has limited its role to be incorporated into societal guidelines.

### Limitations

Our study's retrospective cohort design and lack of randomization present inherent limitations. We included patients with documented DVT from 4 Michigan hospitals. A key limitation was the reliance on data manually entered into the EMR, occasionally leading to discrepancies. While EMR data offer valuable insights, potential limitations inherent to this source include incomplete documentation and coding inconsistencies.

The chronicity of DVT was defined in our methodology by societal guidelines of acute, subacute, and chronic. The authors decided to use ultrasound characteristics to define chronicity vs symptom onset. The timing of the DVT is often erroneous due to delayed patient presentation and onset of symptoms. There were also inherent limitations in using ultrasound data as there were different vascular surgeons, cardiologists, and radiologists interpreting the ultrasounds.

The length of stay has a SD of 5.5 days which is reflective of the wide range of comorbidities affecting this patient population and the different patterns of inpatient management across the 3 specialties.

There was an association not causation between IVUS and increased rates of venoplasty and stenting, which is related to a selection bias. For example, the most aggressive operators may believe in positive outcomes with MT and these operators are also individuals who lead the most modern practices, which typically include 100% IVUS use.

### Clinical implications and future perspectives

The integration of IVUS in venous interventional procedures has significant clinical implications. It enhances the safety and efficacy of the procedures and contributes to better long-term outcomes for patients with venous obstruction. Future research should focus on establishing standardized IVUS-guided protocols and exploring IVUS utility in various venous pathologies, including acute and chronic DVT. IVUS with other advanced imaging techniques in the rapidly evolving endovascular intervention represents a promising new frontier. This imaging-based interventional paradigm shift may significantly enhance the comprehensiveness and accuracy of vascular assessments, ultimately improving patient outcomes.

## Conclusion

Our study underscores the efficacy of MT in restoring venous flow in patients with DVT. This treatment modality is utilized across multiple medical specialties, highlighting its versatility in clinical practice. Furthermore, the study revealed extensive use of IVUS in conjunction with MT. The association of IVUS with increased rates of stent placement and venoplasty suggests its potential role in enhancing clinicians' understanding of venous anatomy. It is important to consider that the small nonrandomized sample size and the high utilization rate of IVUS might limit the generalizability of these findings and the limitation warrants cautious interpretation. Therefore, it is imperative in the future to create randomized control trials for MT with adjunctive IVUS usage for the treatment of DVT to develop societal guidelines in the venous space.
